# Prevalence, Gender and Age Differences of Dating Aggression Among Italian Adolescents

**DOI:** 10.5964/ejop.v16i4.1822

**Published:** 2020-11-27

**Authors:** Gaia Cuccì, K. Daniel O’Leary, Maria Giulia Olivari, Emanuela Confalonieri

**Affiliations:** a CRIdee, Department of Psychology, Università Cattolica del Sacro Cuore, Milano, Italy; b Department of Psychology, Stony Brook University, Stony Brook, NY, USA; University of Liverpool, Liverpool, United Kingdom

**Keywords:** adolescence, dating aggression perpetration, dating aggression victimization, gender differences, age differences

## Abstract

The present study represents an effort to expand and deepen the scant literature on Adolescent Dating Aggression (ADA) within the Italian context; adolescent dating aggression is a public health issue of interest due to its increasing frequency among adolescents. The prevalence of verbal-emotional and physical ADA was examined as well as gender and age differences in a sample of Italian adolescents. Participants included 436 adolescents (47.7% males; 52.3% females) living in northern Italy, aged 16 to 18 years (M = 17.11). Participants completed the Conflict in Adolescent Dating Relationships Inventory measuring abusive behaviors between adolescent dating partners. Non-parametric analyses were computed. Verbal-emotional ADA perpetration and victimization were much more common than physical ADA perpetration and victimization. Females reported higher levels of verbal-emotional and physical ADA perpetration than males. To fully investigate gender differences single behaviors were analyzed and described. Finally, age differences emerged only for perpetrated verbal-emotional abuse with such aggression being highest at age 18. This research suggests that in order to prevent the onset of dating aggression in teens in northern Italy, prevention programs may need to begin earlier than previously provided in junior high school. Another core conclusion is that physical aggression against partners is a problem for both males and females, thus intervention for the empowerment of interpersonal skills are needed.

Adolescence Dating Aggression (ADA) may be defined as a type of intimate partner violence that occurs between two adolescents in a close relationship (Centers for Disease Control and Prevention, 2016). It is believed that dating aggression in adolescence is due to immaturity and lack of experience, together with more adult-like efforts to dominate and control one’s partner, may lead an adolescent to resort to aggressive behaviors which do not represent fully adult-like pattern of violence (Wolfe et al., 2001a, 2005).

Studies on dating aggression (Cuccì, O’Leary, Olivari, Bonanomi, & Confalonieri, 2019; González & Santana, 2001; Hird, 2000; Jackson, Cram, & Seymour, 2000; Katz, Carino, & Hilton, 2002; Muñoz-Rivas, Graña, O’Leary, & González, 2007a, 2007b; Olivari, Cuccì, & Confalonieri, 2017) have identified several types of aggressive behaviors that may occur in relationships. Psychological aggression (both verbal and emotional abusive behaviors such as name calling, shaming, bullying, embarrassing on purpose) occurs most often, followed by physical aggression (e.g., shoving, hair pulling, slapping, punching), and sexual aggression (e.g., unwanted touching, kissing, sex). In young teens, such forms of aggression tend to increase over time (Arriaga & Foshee, 2004; Champion, Foley, Sigmon-Smith, Sutfin, & DuRant, 2008; Nocentini, Menesini, & Pastorelli, 2010) showing an escalation from minor forms of aggression (e.g., verbal-emotional violence) to more severe ones (e.g., physical or sexual violence).

ADA is associated with several risk-taking behaviors such as risky sexual behaviors and substance abuse (Ackard, Neumark-Sztainer, & Hannan, 2003; Bonomi, Anderson, Nemeth, Rivara, & Buettner, 2013) and with many negative outcomes, such as eating disorders, depressive symptoms, suicidal thoughts, stress, anxiety, lack of self-esteem, and lower school achievement (Ackard et al., 2003; Bonomi et al., 2013; Callahan, Tolman, & Saunders, 2003; Holt & Espelage, 2005). Many studies have also shown that ADA may affect the way adolescents and young adults establish subsequent relationships (Wolfe et al., 2001b) and be a precursor to aggression towards a partner later and in more stable relationships, including marriage (Exner-Cortens, Eckenrode, & Rothman, 2013; Gómez, 2011; Hamby, Finkelhor, & Turner, 2012; Smith, White, & Holland, 2003; O’Leary, Tintle, & Bromet, 2014; Temple, Shorey, Fite, Stuart, & Le, 2013).

Studies focusing specifically on ADA prevalence were carried out in USA (Foshee & Matthew, 2007; Foshee et al., 2009; O’Leary, Slep, Avery-Leaf, & Cascardi, 2008), New Zealand (Jackson et al., 2000) and in Europe (United Kingdom: Hird, 2000; Windle & Mrug, 2009; Spain: Muñoz-Rivas et al., 2007b; Italy: Nocentini et al., 2010) demonstrating that psychological aggression is the most prevalent form of ADA, while sexual aggression was the least prevalent (Foshee & Matthew, 2007; Foshee et al., 2009; Hird, 2000; Jackson et al., 2000).

As for gender differences in aggressive behaviors, studies on ADA (e.g., Giordano, Soto, Manning, & Longmore, 2010; Lewis & Fremouw, 2001; Offenhauer & Buchalter, 2013; Swahn et al., 2008) generally indicate that males and females are relatively equally likely to perpetrate such form of aggression in terms of frequency. However, the literature is characterized by a heated debate concerning the reasons behind the aggressive behaviors. According to White and Kowalski (1994), the notion of “the less aggressive” woman is a socio-historical myth. The stereotype of gender differences in aggression has sustained men in maintaining a position of power over women. The authors noted that, although women are reported to commit fewer crimes than men do, this does not imply that they are not aggressive. This general misperception could be due to the fact that the situations in which women will display aggressive behaviors appear to be more circumscribed. Traditionally, gender roles relegate women to the domestic sphere and there was little opportunity to engage in violence in the public sphere. Conversely, men are more likely than women to express their aggression publicly and physically. In line with these considerations, feminist theorists have often contended violence perpetrated by women as acts of self-defense (e.g., Hettrich & O’Leary, 2007; Stuart et al., 2006). Moreover, aggressive behaviors perpetrated by women are qualitatively different because these acts usually do not cause fear or injuries (Dobash, Dobash, Wilson, & Daly, 1992), thus this theory highlights female power imbalance and emotional devaluation. Though theses issue also are still debated in literature.

Among adolescents, as previously stated, aggressive behaviors within the romantic couple are perpetrated by both males and females. Within the branch of research focusing specifically on gender differences in ADA, results are quite heterogeneous. Most of the studies reported higher rates of physical ADA perpetration for females than males, and males generally reported being victims of physical ADA more frequently than females. However, with regards to severity, males generally reported no physical injury whereas females suffered more severe consequences by male partners who perpetrated more severe forms physical aggression (Foshee et al., 2009; Muñoz-Rivas et al., 2007a, 2007b; Windle & Mrug, 2009). In particular, O’Leary and colleagues (2008) found that both male and female adolescents reported engaging in physical aggression more than experiencing such form of aggression as victims. Further, when physical aggression occurred, it was largely bidirectional (i.e., both partners were aggressive). In addition, those adolescents who were engaged to be married reported the highest rates of physical aggression. Conversely, a United Kingdom study (Hird, 2000) showed that girls reported higher rates of perpetration and victimization for both psychological and physical ADA, while in New Zealand, similar percentages of psychological, physical and sexual ADA were observed for both males and females (Jackson et al., 2000).

As for age differences, studies presented quite different findings probably due to methodological variability. A cross-sectional study carried out in Spain with adolescents and young adults aged 16-20 years, showed very high rates of psychological ADA that was stable across the age groups. Conversely, rates of physical ADA decreased significantly across the age groups, while health consequences resulting from aggression became more severe with age (Muñoz-Rivas et al., 2007b). On the contrary, longitudinal studies examining growth trajectories of ADA observed different patterns. O’Leary and Slep (2003) observed a stable pattern for physical ADA from the 10th grade to the 12th grade, while Wolfe and colleagues (2003) found that the mean trajectories for physical and psychological ADA were characterized by a steady decrease from ages 14–16 to ages 16–18. A study by Foshee and colleagues (2009) showed that the development of physical and sexual ADA perpetration decreased from ages 13 to 19, with peak ages ranging from 16 to 17 years of age. As for psychological ADA perpetration, authors observed an increasing pattern from ages 13 to 19.

Concerning Italy, ADA is emerging as a public health issue. A survey carried out by Telefono Azzurro and DoxaKids (2014) with approximately 1500 Italian male and female adolescents attending junior high school and high school, reported that 8% were threatened by a romantic partner; 33% were victims of psychological ADA; 6% were physically beaten at least once by a romantic partner, and 6% were forced to have sex.

To date, research on ADA in Italy specifically focused on relationship’s characteristics (e.g., support, power imbalance, conflict). Menesini and Nocentini (2008) found that with age relationships became gradually more supportive, but also more conflicted. In fact, in late adolescence, relationships are more characterized by expectations, emotional commitment and intimacy which may lead partners to overreact thereby increasing the intensity of the conflict (Seiffge-Krenke, 2003).

A cross-cultural research, carried out in Italy and Canada (Connolly et al., 2010), showed that levels of conflict were associated with ADA in both countries, while power imbalance uniquely in Italy. Finally, Menesini, Nocentini, Ortega-Rivera, Sanchez, and Ortega (2011) in another cross-cultural study comparing adolescents from Italy and Spain, found that in both countries couples experiencing reciprocal psychological and physical ADA were more likely to have higher levels of couple conflict and power imbalance. However, Italian literature investigating ADA prevalence, gender and age differences is scarce. The only longitudinal study carried out in a small city in the center of Italy, found that moderate physical ADA showed an average decreasing trend from 16 to 18 years (Nocentini et al., 2010).

Our work represents an effort to expand and deepen the scarce existing literature on ADA in the Italian context, by employing a wide sample, in order to deepen aspects that, to date, are quite unexplored. It should be also considered that ADA is an emerging and increasing health problem within the Italian context, thus it seems important to expand the knowledge base on this sensitive issue.

Therefore, the aims of this study are to: a) describe the prevalence of ADA in a sample of Italian adolescents; b) evaluate gender differences both in relation to each single aggressive behavior and two types of ADA (verbal-emotional and physical) perpetration and victimization. Going through each single behaviors will allow a better understanding of the nature of the phenomenon and to identify which are the most frequent behaviors perpetrated by adolescents, in order to catch the possible meanings and functions that ADA may play; c) evaluate age differences in relation to two types of ADA (verbal-emotional and physical) perpetration and victimization.

No hypothesis will be formulated for the first aim, since its descriptive in nature. As for the second aim, in line with previous findings, we expect to find higher rates of verbal-emotional and physical ADA perpetration among female than male adolescents (Foshee et al., 2009; Muñoz-Rivas et al., 2007a, 2007b; Windle & Mrug, 2009) and to find higher rates of verbal-emotional and physical ADA victimization among male than female adolescents (Fernández-González, O’Leary, & Muñoz-Rivas, 2014). As for the third aim, we expect to find stable rates of psychological ADA across the age groups and decreasing rates of physical ADA across the age groups (Muñoz-Rivas et al., 2007b).

## Method

### Participants and Procedure

Participants were 859 adolescents (36.5% males; 63.5% females) living in northern Italy (Lombardy and Piedmont) aged 16 to 18 years (*M* = 17.01 years, *SD* = 0.8 years). Within this sample 709 participants (83.3%) were adolescents who have already experienced a romantic relationship (currently or in the past). Starting from this sample, a subsample balanced for gender was randomly extracted, using SPSS (Version 18). Therefore, the final sample included 436 adolescents (47.7% males; 52.3% females) aged 16 to 18 years (*M* = 17.11 years, *SD* = 0.8 years). The average age was 17.18 years for males (*SD* = 0.8 years) and 17.05 years for females (*SD* = 0.8 years).

There are three types of high school programs students in Italy can select from: one that is more theoretically oriented (a school whose aim is to prepare students to enter university), another that is more professionally oriented (a school whose aim is to prepare students for employment after high school), one that is more technically oriented (a school whose aim is prepare students to enter technical schools). Fifty percent of the participants attended a theoretical program, 32.3% attended a professional program, and 17.7% attended a technical program.

Approval for the study was obtained from the Ethical Commission of the Università Cattolica del Sacro Cuore of Milan. The headmasters and class teachers of the involved schools provided permission for the study, and parents provided written consent for their children’s participation. The teenage participants also provided their consent.

### Instruments

For this study, a specific assessment self-report instrument with different formats was administered in person:

a) A series of questions with various response options that assessed relevant information concerning:

- Demographics, e.g., age, sex, nationality, type of school attended.
- Dating relationship variables, e.g., whether they have ever experienced a romantic relationship or not, type of relationship (current vs. past), duration of the relationship, age of the romantic partner, perceived importance of the relationship).
- Sexual behaviors variables, e.g., whether they have ever had a sexual intercourse, age of the first sexual intercourse).

b) A short version of the Conflict in Adolescent Dating Relationships Inventory (CADRI; Wolfe et al., 2001a). The original version is a 35-item instrument to assess multiple forms of abusive behaviors that may occur between adolescent dating partners. The instrument showed fair psychometric properties for verbal-emotional (α = .81; Wolfe et al., 2001a) and physical abuse perpetration and victimization (α = .76; Wolfe et al., 2001a). Response choices for each item were measured on a 4-point step scale (1 = *never* to 4 = *often*). An interpreter translated the items of the CADRI from English into Italian and the items were preliminary administered to 100 adolescents in a pilot study. Then, a native English speaker performed the back translation. In the present study, we employed only an adaptation of two subscales: verbal-emotional abuse perpetration and victimization (9 items; e.g., *“I insulted her/him with put-downs”*, respectively α = .79; α = .80) and physical abuse perpetration and victimization (4 items; e.g., *“I kicked, hit or punched her/him”*, respectively α = .73; α = .82). Items scores are summed for the score for each subscale, as suggested by the authors (Wolfe et al., 2001a).

## Results

### Dating Behavior

Among participants, 61.6% reported that they were experiencing a romantic relationship at the time of the study and 38.4% had experienced a romantic relationship in the past. The mean age of the romantic partner was 17.73 (*SD* = 0.8 years) and no significant differences emerged between male and female participants in terms of age of partner, *t*(418) = -1.5; *p* = .127. The mean duration of the relationships was about 10.15 months (*SD* = 9.54), with significant differences between sexes, *t*(425) = -2.3; *p* = .017, with the females (*M* = 11.20 months; *SD* = 10.4) maintaining their relationships for longer periods of time than males (*M* = 9 months; *SD* = 8.4). Among participants in a current relationship, males described their relationship as very important (63.4%), quite important (35.5%) and not very important (1.1%). Female participants described their relationship as very important (83.2%) and quite important (16.8%), χ^2^(2) = 11.64; *p* = .003. Among participants who experienced a relationship in the past, males described it as very important (21.5%), quite important (70.5%), and not very important (8%). Female participants described their past relationship as very important (43.1%), quite important (50%), and not very important (6.9%), χ^2^(2) = 11.72; *p* = .003.

### Prevalence of Verbal-Emotional and Physical ADA Perpetration and Victimization

Firstly, at a descriptive level, frequency analysis was carried out to report the percentages of adolescents who have perpetrated or experienced at least once verbal-emotional or physical abuse. Percentages of adolescents reporting to have perpetrated at least one act (i.e., participants who scored from 2 to 4) of verbal-emotional abuse was 96.1% while physical abuse was 30.7%. Percentages of adolescents reporting to have experienced at least one act (i.e., participants who scored from 2 to 4) of verbal-emotional abuse was 97.9% while for physical abuse it was 32.1%.

Parametric assumptions were in fact verified for each item and since parametric assumptions were violated, non-parametric analyses were conducted. The Wilcoxon signed-rank test was calculated to describe the prevalence of ADA within our sample. Analyses showed that the median score of verbal-emotional abuse perpetration (*Mdn* = 2.00) was significantly higher than physical abuse (*Mdn* = 1.00; *Z* = -16.959; *p* < .001). The same result held for the mean score of verbal-emotional abuse victimization (*Mdn* = 2.00), which was significantly higher than physical abuse (*Mdn* = 1.00; *Z* = -17.440; *p* < .001).

### Verbal-Emotional and Physical ADA Perpetration and Victimization: Gender Differences

To assess gender differences regarding each single ADA behavior, Chi Square tests were calculated on CADRI item describing Verbal Emotional Abuse and Physical Abuse perpetration and victimization. Because multiple comparisons were made, a Bonferroni procedure was used to reduce the risk of inflating the Type I error rate. The significance of each Chi Square test was evaluated at the .005 level (.05/10).

As shown in Table 1, the most commonly perpetrated behaviors among females were making the partner feel jealous, bringing up something from the past, keeping track of the partner’s movements, and accusing the partner of flirting with another person. Making fun of the partner in front of friends was more frequent among males.

**Table 1:** Reports of Perpetration of Verbal Emotional Abuse

Behavior	Male (%)	Female (%)	χ^2^
I did something to make him/her feel jealous	68.3	77.2	4.388*
I brought up something bad that he/she had done in the past	56.7	69.3	7.395**
I said things just to make him/her angry	66.8	68.9	n.s.
I spoke to him/her in a hostile or mean tone of voice	72.1	77.2	n.s.
I insulted him/her with put-downs	67.3	68.9	n.s.
I ridiculed or made fun of him/her in front of others	27.9	16.7	7.972**
I kept track of who he/she was with and where he/she was	66.8	76.3	4.836*
I blamed him/her for the problem	66.8	73.7	n.s.
I accused him/her of flirting with another girl/boy	44.2	54.8	4.883*

*Note.* Each of the Chi Square test was evaluated at the .01 significance level to control for family-wise error accompanying multiple comparisons.

*p < .05. **p < .01.

As reported in Table 2, percentages of physical perpetrated behaviors were generally higher among females. In particular, female participants reported significantly higher rates of engaging in the following behaviors: throwing something at their partner and slapping their partner, or pulling their partner’s hair.

**Table 2:** Reports of Victimization of Verbal Emotional Abuse

Behavior	Male (%)	Female (%)	χ^2^
He/she did something to make me feel jealous	68.3	77.2	4.388*
He/she brought up something bad that I had done in the past	55.8	59.2	n.s.
He/she said things just to make me angry	67.3	70.6	n.s.
He/she spoke to me in a hostile or mean tone of voice	73.1	75.0	n.s.
He/she insulted me with put-downs	65.9	71.5	n.s.
He/she ridiculed or made fun of me in front of others	33.2	22.4	6.366*
He/she kept track of who I was with and where I was	69.7	76.8	n.s.
He/she blamed me for the problem	76.9	69.3	n.s.
He/she accused me of flirting with another girl/boy	50.0	51.3	n.s.

*Note.* Each of the Chi Square test was evaluated at the .01 significance level to control for family-wise error accompanying multiple comparisons.

*p < .05.

As shown in Table 3 for ADA verbal-emotional victimization, female participants reported their partners made them feel jealous more frequently than male participants. The male participants reported more their partners made fun of them in front of friends more frequently than female participants.

**Table 3:** Reports of Perpetration of Physical Abuse

Behavior	Male (%)	Female (%)	χ^2^
I threw something at him/her	14.4	23.2	5.493*
I kicked, hit or punched him/her	13.5	20.2	n.s.
I slapped him/her or pulled his/her hair	9.6	19.3	8.143**
I pushed, shoved, or shook him/her	15.8	19.7	n.s.

*Note.* Each of the Chi Square test was evaluated at the .01 significance level to control for family-wise error accompanying multiple comparisons.

*p < .05. **p < .01.

Finally, as shown in Table 4 for ADA physical victimization, male participants reported that their partner threw something at them, slapped them or pulled their hair, and pushed, shoved, or shook them more frequently than female participants.

**Table 4:** Reports of Victimization of Physical Abuse

Behavior	Male (%)	Female (%)	χ^2^
He/she threw something at me	23.1	12.3	8.809**
He/she kicked, hit or punched me	25.0	10.1	16.983***
He/she slapped me or pulled my hair	24.5	7.9	22.567***
He/she pushed, shoved, or shook me	22.6	13.6	5.997**

*Note.* Each of the Chi Square test was evaluated at the .01 significance level to control for family-wise error accompanying multiple comparisons.

**p < .01. ***p < .001.

To assess gender differences regarding the two types of ADA (verbal-emotional and physical), Mann-Whitney test was calculated considering the two CADRI subscales for both perpetration and victimization. Gender differences emerged for perpetrated verbal-emotional abuse (*U* = 18460; *p* < .001) with females reporting higher levels of aggression (*Mdn* = 2.11) than males (*Mdn* = 2.00). The same was for perpetrated physical abuse (*U* = 19910; *p* = .001), with females reporting higher levels of aggression (*Mdn* = 1.50) than males (*Mdn* = 1.00).

No significant differences emerged for the subscales assessing victimization behaviors.

### Verbal-Emotional and Physical ADA Perpetration and Victimization: Age Differences

Kruskal-Wallis H tests were carried out considering each of the two CADRI subscales for both perpetration and victimization as dependent variables, and age (divided in three age classes, respectively 16, 17 and 18-year-old adolescents) as the independent variable. Participants for each class were respectively: 118, 151, 167.

Analyses demonstrated significant differences only for perpetrated verbal-emotional abuse, χ^2^(2) = 8.125, *p* = 0.017. Pairwise post hoc comparisons indicated that 16-year-old participants reported a significantly lower mean rank score (195.36) than 18-year-old males (238.01). No significant differences emerged for perpetrated physical abuse and for verbal-emotional and physical victimization.

## Discussion

Since ADA is an emerging and increasing health problem in Italy and the existing literature on ADA in the Italian context is quite scarce, this study aimed at expanding the knowledge base on this sensitive issue. Our work had three different aims.

With the first one we aimed at providing a description of the frequency of ADA in a sample of Italian adolescents at a descriptive level. Our findings confirmed the presence of ADA among Italian dating adolescents, as stated by previous research (e.g., Nocentini et al., 2010; Ortega et al., 2010). In our study, in particular, it emerged that 96% of the participants has perpetrated at least one act of verbal-emotional abuse and around 30% of the sample has engaged in physically aggressive behaviors towards their romantic partner at least once. We believe that these data are important because they suggest that adolescents involved in a romantic relationship, even if rarely, may resort to dysfunctional interaction strategies to deal with the partner.

Around 98% of the sample experienced at least one act of verbal-emotional abuse victimization and 32% of the sample reported experiencing physical abuse victimization at least once. These findings are in line with previous evidence emerged in Italy (e.g., Nocentini et al., 2010; Ortega et al., 2010) and Spain (Muñoz-Rivas et al., 2007b). These data are particularly significant when considering that more than 60% of the study participants described their relationships as important, meaning that adolescent participants consider their relationships, even if partially characterized by aggression and conflict, as significant for themselves. This may indicate that these aggressive behaviors are becoming more common within adolescent romantic relationships. On the contrary, results are quite different from the ones emerged in the survey carried out by Telefono Azzurro and DoxaKids (2014) that showed higher percentages for both verbal-emotional and physical ADA. This difference could be a function of using different measure use to asses ADA. In the above-mentioned survey ADA behaviors were measured using *ad hoc items* while in the present study we employed the CADRI inventory (Wolfe et al., 2001a). Another possible explanation could be linked to the age of participants: our sample consisted of adolescents aged 16-18 years while no information was available on the exact age range of participants responding to the previous survey conducted in Italy.

As for the prevalence of the two types of ADA perpetration and victimization, in our study it emerged that verbal-emotional abuse was much more common than physical abuse among adolescent participants. Our participants appear to both perpetrate and experience such abuse, as was observed in other studies (Foshee & Matthew, 2007; Foshee et al., 2009; Hird, 2000; Jackson et al., 2000).

The second aim of our study was to evaluate the gender differences starting from the analyses of each single aggressive behavior, which allowed us to understand the nature of the aggressive behaviors in greater detail. Moreover, we investigated gender differences across the two types of ADA (verbal-emotional and physical) perpetration and victimization, since in Italian literature little attention has been paid on gender differences across the two types of ADA.

As for single-item analysis, regarding the perpetration of verbal-emotional aggression, girls tend to express their aggression though behaviors of jealousy and control (e.g., *“I did something to make him/her feel jealous*; *I kept track of who he/she was with and where he/she was”*), that may be an expression of feelings of personal insecurity towards the self or towards the future of the relationship. Boys, on the other hand, seem to engage more in behaviors involving the social domain (e.g., *“I ridiculed or made fun of him/her in front of others”*), this may be in order to establish or maintain their social status within the peer context.

Regarding the victimization of verbal-emotional ADA, in a specular way adolescents reported to be victim of the same behaviors they also reported to perpetrate more, suggesting the potential development of regular dysfunctional pattern of interaction within the couple. These behaviors may be perpetrated, indeed, as forms of revenge or imitation between the partners where each partner use the same behaviors to get even with the partner.

Regarding the perpetration of physical aggression, girls tend to engage more than their male peers in moderate aggressive actions, such as slapping and pulling the hair of the partner. However, the severity as well as the consequences of these physical injuries are less severe, as shown in previous studies where male partners perpetrated more severe forms physical aggression (e.g., Cuccì et al., 2019; Foshee et al., 2009; Muñoz-Rivas et al., 2007a, 2007b; Windle & Mrug, 2009). Regarding physical aggression victimization, despite the low rates of frequency, for each behavior, male participants reported to be victimized more frequently than female participants. A possible explanation could be that, within the context of a heated situation (e.g., conflict within the couple), a lack of skills in female emotion regulation may resulted in ADA as an expression of emotional discomfort rather than a real intention to physically harm the partner.

These findings, indeed, support the idea that ADA may develop as a dysfunctional strategy of interaction between the two partners.

Concerning gender differences in relation to the two types of ADA (verbal-emotional and physical) perpetration and victimization, girls engaged more frequently than boys in verbal-emotional and physical ADA perpetration. These findings confirm previous evidence (Foshee et al., 2009; Muñoz-Rivas et al., 2007a, 2007b; O’Leary et al., 2008; Windle & Mrug, 2009) showing higher rates of perpetration among female adolescents. These findings could be interpreted considering some relationship’s characteristics. The length of the relationship was, indeed, significantly higher for females and that most of them described the relationship as very important. This may imply that girls feel to be more invested in their relationships. Therefore, it is possible to hypothesize that female adolescents resort to dysfunctional behaviors to maintain and protect their romantic relationships, not being able to engage in more adaptive conflict resolution strategies. Our findings are in line with the developmental perspective that female adolescent aggression toward their partner may result from their relative inexperience in managing romantic relationships. This lack of skill would lead females to adopt more masculine aggressive behaviors to solve their conflicts with the romantic partner, as showed in previous studies (Connolly et al., 2015; O’Leary et al., 2008; Rose & Rudolph, 2006; Wolfe, Scott, & Crooks, 2005). As suggested by some feminist theorists a small but significant percentage of females may also resort to violence as an act of self-defense to contrast male predominance in power (Hettrich & O’Leary, 2007; Stuart et al., 2006; White & Kowalski, 1994). Female adolescents may also resort aggressive behaviors as an effort to restore balance when perceiving an imbalance of power in their relationships. Previous studies have demonstrated that these characteristics are associated with ADA (Connolly et al., 2010; Menesini & Nocentini, 2009). At the same time, it is possible to consider that females engage more frequently in mutual aggressive relationships. This may mean that, within the romantic couple, partners tend to use aggressive behaviors as a form of interaction with each other. Thus, it is possible that partners learn dysfunctional strategies, modeling their behaviors by observing each other (Bandura, 1977).

Finally, it is important to consider that gender differences may also be due to social desirability bias, (i.e., the tendency of people to deny or underreport behaviors that are socially unacceptable). Males may indeed find it difficult to admit they acted physically aggressive towards their female partner, while it might be easier to report ADA victimization. This may also explain why no significant differences were found for ADA victimization, differently from what we expected. As Fernández-González and colleagues (2014) reported, both males and females are less likely to report negative behaviors about themselves than about their partners. With a large sample of adolescents from Madrid (~850), social desirability had a small but significant association with reports of ADA, but covariance corrections for social desirability did not alter the conclusions about such aggression. Because of the small correlation of social desirability with perpetration and victimization for females (-.19, -.16) and males (-.10, -.11), the corrections for social desirability did little to alter any overall conclusions based on uncorrected means. Using uncorrected or corrected means for social desirability, males engage in more sexual ADA and females engage in more psychological and physical ADA. If one wants to obtain the maximal prevalence rates, Fernández-González and colleagues (2014) found that maximal dyadic reports (based on either the self or partner) significantly increased the prevalence rates of aggression. However, the overall conclusions about perpetration and victimization did not differ. If one wishes to estimate maximal rates of partner aggression but one only has self-reports, Heyman and Schlee (1997), and O’Leary and Williams (2006) have used correction factors with married couples and such an approach could be used with dating couples.

Our third aim was to evaluate age differences in relation to the two types of ADA (verbal-emotional and physical) perpetration and victimization. Findings in the literature are mainly focused on ADA perpetration and victimization, and such findings have been mixed. In line with previous cross-sectional research, we expected to find stable rates of psychological ADA across the age groups and decreasing rates of physical ADA across the age groups (Muñoz-Rivas et al., 2007b). Our results showed, on the contrary, that the different aggressive behaviors were stable across the ages sampled. Significant differences emerged only for ADA verbal-emotional perpetration with 18-year-old adolescents reporting higher rates of such aggression than 16-year-old participants. This different result could be due to the fact that we employed a smaller range of age (16-18) than the one of Muñoz-Rivas and colleagues (16-20). Moreover, no significant decrease emerged for physical ADA perpetration and victimization. In our sample physical ADA perpetration and victimization rates seemed to remain stable across ages, similar to what was observed by O’Leary and Slep (2003) in their longitudinal study, carried out within the short range of time of three months. Conversely, other longitudinal studies on physical ADA showed a decrease over time (Foshee et al., 2009; Wolfe et al., 2003). In particular, our results differ from Nocentini and colleagues’ (2010) findings on a sample of 181 Italian adolescents reporting an average decreasing trend from 16 to 18 years. Moreover, our results showing an increasing rate for verbal-emotional ADA perpetration, are in line with the fact that among our participants verbal-emotional aggression was much more common than physical abuse. Finally, it is possible that this difference could be due to methodological reasons: the research design (cross-sectional vs. longitudinal) and the measure use to assess ADA (CADRI vs. Conflict Tactics Scale [CTS2]). The choice of instrument should be considered when comparing results of different studies since the two scales measure psychological and physical dating aggression via different behaviors. In particular the CTS2 (Straus, Hamby, Boney-McCoy, & Sugarman, 1996), includes more severe acts of physical ADA compared with the CADRI, such as beating, choking, burning, and scalding a partner (Cascardi, Blank, & Dodani, 2019). Moreover, though developed with a similar goal, the assessment of psychological and physical abuse towards a romantic partner is different, since CADRI was developed specifically for adolescent samples (Cascardi, Blank, & Dodani, 2019; Wolfe et al., 2001a).

Our findings need to be evaluated with recognition of some limitations. The cross-sectional design, as in other previous studies on ADA (Muñoz-Rivas et al., 2007a, 2007b), limits inferences about the developmental changes. Longitudinal studies could help explain the development across time of this phenomenon. There are very few longitudinal studies that used the same measures of aggression. This need for longitudinal research seems crucial, especially since we have almost no research beginning in the teen years and extending into long-term adult relationships or marriage. Such research is needed to learn which men and women cease their aggression and which continue or escalate their aggressive behaviors.

Moreover, in this study, we did not investigate participants’ sexual orientation, thus we do not know if they reported ADA occurring in heterosexual or same-sex relationships. Future studies may consider this aspect to obtain more generalizable results. Finally, the CADRI (Wolfe et al., 2001a) is a frequency-based measure of ADA, thus this scale does not investigate motivations for ADA. In the future, qualitative studies, that allow a better understanding of perceptions and attitudes towards a phenomenon (e.g., Olivari, Confalonieri, & Ionio, 2011), are warranted in order to gain more information to better understand the reasons linked to ADA. Future research should be also aimed at collecting dyadic data in order to assess mutuality of ADA within the same couple in detail, considering relationship characteristics such as relationship length, partner age and balance of power.

We believe our work could provide interesting clues for ADA prevention in Italian high schools. It is clear that both male and female adolescents engage in ADA, so programs should target both male and female aggression. Additionally, interventions should begin in high school or earlier since ADA behaviors are already observed among 16-year-old adolescents. Finally, considering that emotional-verbal and psychological abuse are the most common forms of ADA, programs should also target these forms of aggression, as to date, the majority of attention on aggression has been devoted to physical and sexual forms. This change in focus may improve adolescent awareness and knowledge on all forms of ADA and their consequences. Finally, it is important to develop not only prevention interventions, but also programs of skills empowerment aimed at making adolescents better able to manage couple conflict and improving positive and adaptive interaction strategies.

## References

[r1] Ackard D. M. Neumark-Sztainer D. Hannan P. (2003). Dating violence among a nationally representative sample of adolescent girls and boys: Associations with behavioral and mental health. The Journal of Gender-Specific Medicine, 6(3), 39–48. 14513575

[r2] Arriaga X. B. Foshee V. A. (2004). Adolescent dating violence: Do adolescents follow in their friends’, or their parents’, footsteps? Journal of Interpersonal Violence, 19(2), 162–184. 10.1177/0886260503260247 15006000

[r3] Bandura, A. (1977). *Social learning theory*. Englewood Cliffs, NJ, USA: Prentice-Hall.

[r4] Bonomi A. E. Anderson M. L. Nemeth J. Rivara F. P. Buettner C. (2013). History of dating violence and the association with late adolescent health. BMC Public Health, 13, 821. 10.1186/1471-2458-13-821 24015863 PMC3847300

[r5] Callahan M. R. Tolman R. M. Saunders D. G. (2003). Adolescent dating violence victimization and psychological well-being. Journal of Adolescent Research, 18(6), 664–681. 10.1177/0743558403254784

[r6] Cascardi M. Blank S. Dodani V. (2019). Comparison of the CADRI and CTS2 for measuring psychological and physical dating violence perpetration and victimization. Journal of Interpersonal Violence, 34(16), 3466–3491. 10.1177/0886260516670182 27760876

[r7] Centers for Disease Control and Prevention. (2016). *Understanding teen dating violence* (Fact-sheet). Retrieved from https://stacks.cdc.gov/view/cdc/38280/cdc_38280_DS1.pdf

[r8] Champion H. Foley K. L. Sigmon-Smith K. Sutfin E. L. DuRant R. H. (2008). Contextual factors and health risk behaviors associated with date fighting among high school students. Women & Health, 47(3), 1–22. 10.1080/03630240802132286 18714709

[r9] Connolly J. Baird K. Bravo V. Lovald B. Pepler D. Craig W. (2015). Adolescents’ use of affiliative and aggressive strategies during conflict with romantic partners and best-friends. European Journal of Developmental Psychology, 12(5), 549–564. 10.1080/17405629.2015.1066244

[r10] Connolly J. Nocentini A. Menesini E. Pepler D. Craig W. Williams T. S. (2010). Adolescent dating aggression in Canada and Italy: A cross-national comparison. International Journal of Behavioral Development, 34(2), 98–105. 10.1177/0165025409360291

[r11] Cuccì G. O’Leary K. D. Olivari M. G. Bonanomi A. Confalonieri E. (2019). Adolescent dating violence perpetration, emotion dysregulation, and parenting styles. Journal of Family Psychology, 33(1), 12–22. 10.1037/fam0000464 30372109

[r12] Dobash R. P. Dobash R. E. Wilson M. Daly M. (1992). The myth of sexual symmetry in marital violence. Social Problems, 39, 71–91. 10.2307/3096914

[r13] Exner-Cortens D. Eckenrode J. Rothman E. (2013). Longitudinal associations between teen dating violence victimization and adverse health outcomes. Pediatrics, 131(1), 71–78. 10.1542/peds.2012-1029 23230075 PMC3529947

[r14] Fernández-González L. O’Leary K. D. Muñoz-Rivas M. J. (2014). Age-related changes in dating aggression in Spanish high school students. Journal of Interpersonal Violence, 29(6), 1132–1152. 10.1177/0886260513506057 24203984

[r15] Foshee V. A. Benefield T. Suchindran C. Ennett S. T. Bauman K. E. Karriker-Jaffe K. J. Mathias J. (2009). The development of four types of adolescent dating abuse and selected demographic correlates. Journal of Research on Adolescence, 19(3), 380–400. 10.1111/j.1532-7795.2009.00593.x 27170829 PMC4861398

[r16] Foshee, V. A., & Matthew, R. (2007). Adolescent dating abuse perpetration: A review of findings, methodological limitations, and suggestions for future research. In D. Flannery, A. Vazonsyi, & I. Waldman (Eds.), *The Cambridge handbook of violent behavior and aggression* (pp. 431–449). New York, NY, USA: Cambridge University Press.

[r17] Giordano P. C. Soto D. A. Manning W. D. Longmore M. A. (2010). The characteristics of romantic relationships associated with teen dating violence. Social Science Research, 39(6), 863–874. 10.1016/j.ssresearch.2010.03.009 21037934 PMC2964890

[r18] Gómez A. M. (2011). Testing the cycle of violence hypothesis: Child abuse and adolescent dating violence as predictors of intimate partner violence in young adulthood. Youth & Society, 43(1), 171–192. 10.1177/0044118X09358313

[r19] González R. Santana J. D. (2001). La violencia en parejas jóvenes [Violence in young couples]. Psicothema, 13, 127–131.

[r20] Hamby S. Finkelhor D. Turner H. (2012). Teen dating violence: Co-occurrence with other victimizations in the national survey of children’s exposure to violence (NatSCEV). Psychology of Violence, 2(2), 111–124. 10.1037/a0027191

[r21] Hettrich E. L. O’Leary D. K. (2007). Females’ reasons for their physical aggression in dating relationships. Journal of Interpersonal Violence, 22(9), 1131–1143. 10.1177/0886260507303729 17704459

[r22] Heyman R. E. Schlee K. A. (1997). Toward a better estimate of the prevalence of partner abuse: Adjusting rates based on the sensitivity of the Conflict Tactics Scale. Journal of Family Psychology, 11(3), 332–338. 10.1037/0893-3200.11.3.332

[r23] Hird M. J. (2000). An empirical study of adolescent dating aggression in the U.K. Journal of Adolescence, 23(1), 69–78. 10.1006/jado.1999.0292 10700373

[r24] Holt M. K. Espelage D. L. (2005). Social support as a moderator between dating violence victimization and depression/anxiety among African American and Caucasian adolescents. School Psychology Review, 34(3), 309–328. 10.1080/02796015.2005.12086289

[r25] Jackson S. M. Cram F. Seymour F. W. (2000). Violence and sexual coercion in high school students’ dating relationships. Journal of Family Violence, 15, 23–36. 10.1023/A:1007545302987

[r26] Katz J. Carino A. Hilton A. (2002). Perceived verbal conflict behaviors associated with physical aggression and sexual coercion in dating relationships: A gender-sensitive analysis. Violence and Victims, 17(1), 93–109. 10.1891/vivi.17.1.93.33641 11991160

[r27] Lewis S. F. Fremouw W. (2001). Dating violence: A critical review of the literature. Clinical Psychology Review, 21(1), 105–127. 10.1016/S0272-7358(99)00042-2 11148892

[r28] Menesini E. Nocentini A. (2008). Comportamenti aggressivi nelle prime esperienze sentimentali in adolescenza. Giornale Italiano di Psicologia, 2, 407–432.

[r29] Menesini E. Nocentini A. (2009). Comportamenti aggressivi nel gruppo dei pari e nelle relazioni sentimentali: quali continuità. Psicologia Clinica dello Sviluppo, 13, 65–81.

[r30] Menesini E. Nocentini A. Ortega-Rivera F. J. Sanchez V. Ortega R. (2011). Reciprocal involvement in adolescent dating aggression: An Italian–Spanish study. European Journal of Developmental Psychology, 8(4), 437–451. 10.1080/17405629.2010.549011

[r31] Muñoz-Rivas M. J. Graña J. L. O’Leary K. D. González M. P. (2007 a). Physical and psychological aggression in dating relationships in Spanish university students. Psicothema, 19(1), 102–107. 17295990

[r32] Muñoz-Rivas M. J. Graña J. L. O’Leary K. D. González M. P. (2007 b). Aggression in adolescent dating relationships: Prevalence, justification and health consequences. The Journal of Adolescent Health, 40(4), 298–304. 10.1016/j.jadohealth.2006.11.137 17367721

[r33] Nocentini A. Menesini E. Pastorelli C. (2010). Physical dating aggression growth during adolescence. Journal of Abnormal Child Psychology, 38, 353–365. 10.1007/s10802-009-9371-8 19957026

[r34] Offenhauer, P., & Buchalter, A. (2013). *Teen dating violence: A literature review and annotated bibliography*. Retrieved from https://www.ncjrs.gov/pdffiles1/nij/grants/235368.pdf

[r35] O’Leary K. D. Slep A. M. S. (2003). A dyadic longitudinal model of adolescent dating aggression. Journal of Clinical Child and Adolescent Psychology, 32(3), 314–327. 10.1207/S15374424JCCP3203_01 12881021

[r36] O’Leary K. D. Slep A. M. S. Avery-Leaf S. Cascardi M. (2008). Gender differences in dating aggression among multiethnic high school students. The Journal of Adolescent Health, 42(5), 473–479. 10.1016/j.jadohealth.2007.09.012 18407042

[r37] O’Leary K. D. Tintle N. Bromet E. (2014). Risk factors for physical violence against partners in the United States. Psychology of Violence, 4(1), 65–77. 10.1037/a0034537 26236543 PMC4520416

[r38] O’Leary K. D. Williams M. C. (2006). Agreement about acts of aggression in marriage. Journal of Family Psychology, 20(4), 656–662. 10.1037/0893-3200.20.4.656 17176201

[r39] Olivari M. G. Confalonieri E. Ionio C. (2011). Italian psychologists’ and midwives’ perceptions of the pregnant teen: A qualitative study. Journal of Reproductive and Infant Psychology, 29(4), 343–353. 10.1080/02646838.2011.631180

[r40] Olivari M. G. Cuccì G. Confalonieri E. (2017). Stile genitoriale autoritario, giustificazione della violenza e messa in atto di comportamenti di Dating Aggression. Maltrattamento e Abuso all’Infanzia*,* 3, 13-30. 10.3280/MAL2017-003002

[r41] Ortega R. Sánchez V. J. Ortega-Rivera J. Nocentini A. Menesini E. (2010). Peer sexual harassment in adolescent girls: A cross-national study (Spain-Italy). International Journal of Clinical and Health Psychology, 10(2), 245–264.

[r42] Rose A. J. Rudolph K. D. (2006). A review of sex differences in peer relationship processes: Potential trade-offs for the emotional and behavioral development of girls and boys. Psychological Bulletin, 132(1), 98–131. 10.1037/0033-2909.132.1.98 16435959 PMC3160171

[r43] Seiffge-Krenke I. (2003). Testing theories of romantic development from adolescence to young adulthood: Evidence of a developmental sequence. International Journal of Behavioral Development, 27(6), 519–531. . 10.1080/01650250344000145

[r44] Smith P. H. White J. W. Holland L. J. (2003). A longitudinal perspective on dating violence among adolescent and college-age women. American Journal of Public Health, 93(7), 1104–1109. 10.2105/AJPH.93.7.1104 12835193 PMC1447917

[r45] Straus M. A. Hamby S. L. Boney-McCoy S. Sugarman D. B. (1996). The revised Conflict Tactics Scale (CTS2). Journal of Family Issues, 17(3), 283–316. 10.1177/019251396017003001

[r46] Stuart G. L. Moore T. M. Gordon K. C. Hellmuth J. C. Ramsey S. E. Kahler C. W. (2006). Reasons for intimate partner violence perpetration among arrested women. Violence Against Women, 12(7), 609–621. 10.1177/1077801206290173 16777948

[r47] Swahn M. H. Simon T. R. Hertz M. F. Arias I. Bossarte R. N. Ross J. G. Hamburger M. E. (2008). Linking dating violence, peer violence, and suicidal behaviors among high-risk youth. American Journal of Preventive Medicine, 34(1), 30–38. 10.1016/j.amepre.2007.09.020 18083448

[r48] Telefono Azzurro & DoxaKids. (2014). “*Osservatorio adolescenti: pensieri, emozioni e comportamenti dei ragazzi di oggi*”. Retrieved from http://www.west-info.eu/it/vamping-e-selfie-le-ossessioni-dei-giovani-italiani/osservatorioadolescenti_report_pdf/

[r49] Temple J. R. Shorey R. Fite P. Stuart G. Le V. (2013). Substance use as a longitudinal predictor of the perpetration of teen dating violence. Journal of Youth and Adolescence, 42, 596–606. 10.1007/s10964-012-9877-1 23187699 PMC3670106

[r50] White J. W. Kowalski R. (1994). Deconstructing the myth of the nonaggressive female: A feminist analysis. Psychology of Women Quarterly, 18(4), 487–508. 10.1111/j.1471-6402.1994.tb01045.x

[r51] Windle M. Mrug S. (2009). Cross-gender violence perpetration and victimization among early adolescents and associations with attitudes toward dating conflict. Journal of Youth and Adolescence, 38, 429–439. 10.1007/s10964-008-9328-1 19636755

[r52] Wolfe, D. A., Scott, K., & Crooks, C. V. (2005). Dating and relationship violence among adolescent girls. In D. Bell-Dolan, E. J. Mash, & S. Foster (Eds.), *Handbook of emotional and behavioral problems in girls* (pp. 381–414). New York, NY, USA: Kluwer Academic. https://doi.org/10.1007/0-306-48674-1_13

[r53] Wolfe D. A. Scott K. Reitzel-Jaffe D. Wekerle C. Grasley C. Straatman A. L. (2001 a). Development and validation of the conflict in adolescent dating relationships inventory. Psychological Assessment, 13(2), 277–293. 10.1037/1040-3590.13.2.277 11433803

[r54] Wolfe D. A. Scott K. Wekerle C. Pittman A. L. (2001 b). Child maltreatment: Risk of adjustment problems and dating violence in adolescence. Journal of the American Academy of Child and Adolescent Psychiatry, 40(3), 282–289. 10.1097/00004583-200103000-00007 11288769

[r55] Wolfe D. A. Wekerle C. Scott K. Straatman A. L. Grasley C. Reitzel-Jaffe D. (2003). Dating violence prevention with at-risk youth: A controlled outcome evaluation. Journal of Consulting and Clinical Psychology, 71(2), 279–291. 10.1037/0022-006X.71.2.279 12699022

